# Diagnostic Stewardship Opportunities for Emergency Department Evaluation of Children with Suspected Urinary Tract Infection

**DOI:** 10.1017/ash.2024.208

**Published:** 2024-09-16

**Authors:** Rachel Wattier, Prachi Singh, Israel Green-Hopkins, Adam Hersh, Andrew Auerbach, Sunitha Kaiser

**Affiliations:** University of California San Francisco; UCSF Benioff Children’s Hospital Oakland; University of Utah

## Abstract

**Background:** Among children who start antibiotics for suspected urinary tract infection (UTI) in emergency departments (EDs), 40-60% have negative urine cultures or other results inconsistent with UTI. Practices contributing to excess antibiotic exposure are not well understood. The goal of this study was to understand diagnostic and post-encounter follow-up processes in children who received antibiotics, in order to define targets for intervention. **Methods:** We identified encounters by children evaluated in two pediatric EDs, over 2 months in the first ED and 9 months in the second ED, to balance different visit volumes. Children 2 months-17 years old were included if they had a urinalysis (UA) and/or urine culture performed, were assigned a primary or secondary diagnosis code for UTI, and initiated antibiotics. Patients were excluded if they received antibiotics prior to the encounter, had prior urologic surgery or device placement, or were immunocompromised or pregnant. Data abstracted by chart review included demographics, documented symptoms, test results, and documented urine culture review and management. Possible UTI symptoms per pediatric criteria included fever, dysuria, urinary frequency, urgency, or hesitancy, suprapubic, abdominal or flank pain, foul smelling urine, or new urinary incontinence. In both EDs, nurses review urine cultures and document changes to treatment plans. Final urine culture results were considered inconsistent with UTI if there was 1) no growth or 2) only mixed growth reported with quantity < 1 00,000 colony forming units/ml. **Results:** Of 150 eligible children, 146 (97%) had at least one UTI symptom and 146 (97%) had abnormal UA **Results:** Urine cultures were not performed in 27 (18%) children. Of 123 encounters with urine cultures performed, 71 (58%) had results inconsistent with UTI. Though 67/71 cultures were marked as reviewed, 43/67 (64%) of the patients who could have stopped antibiotics per guideline recommendations did not have documented plans to stop. In those who had documented plans to stop antibiotics, nurses reached 20/23 (87%) caregivers by phone to communicate these recommendations. **Conclusion:** Many children suspected to have UTI at the time of ED evaluation do not meet criteria for UTI. We found that the most frequent departures from evidence-based practice recommendations were 1) not sending urine cultures, and 2) not stopping antibiotics when culture results did not support the suspected UTI diagnosis. Further investigation should explore barriers and facilitators to these evidence-based practices to develop population- and context-specific diagnostic stewardship strategies.

